# Chromatin Remodeling Protein SMAR1 Is a Critical Regulator of T Helper Cell Differentiation and Inflammatory Diseases

**DOI:** 10.3389/fimmu.2017.00072

**Published:** 2017-02-09

**Authors:** Bhalchandra Mirlekar, Dipendra Gautam, Samit Chattopadhyay

**Affiliations:** ^1^Chromatin and Disease Biology Laboratory, National Centre for Cell Science, Pune, India; ^2^Lineberger Comprehensive Cancer Center, University of North Carolina, Chapel Hill, NC, USA; ^3^Cancer Biology and Inflammatory Disorder Division, Indian Institute of Chemical Biology, Kolkata, India

**Keywords:** asthma, colitis, MAR, regulatory T cells, SMAR1, T helper cells

## Abstract

T cell differentiation from naïve T cells to specialized effector subsets of mature cells is determined by the iterative action of transcription factors. At each stage of specific T cell lineage differentiation, transcription factor interacts not only with nuclear proteins such as histone and histone modifiers but also with other factors that are bound to the chromatin and play a critical role in gene expression. In this review, we focus on one of such nuclear protein known as tumor suppressor and scaffold matrix attachment region-binding protein 1 (SMAR1) in CD4^+^ T cell differentiation. SMAR1 facilitates Th1 differentiation by negatively regulating T-bet expression *via* recruiting HDAC1–SMRT complex to its gene promoter. In contrast, regulatory T (T_reg_) cell functions are dependent on inhibition of Th17-specific genes mainly IL-17 and STAT3 by SMAR1. Here, we discussed a critical role of chromatin remodeling protein SMAR1 in maintaining a fine-tuned balance between effector CD4^+^ T cells and T_reg_ cells by influencing the transcription factors during allergic and autoimmune inflammatory diseases.

## Introduction

Various subsets of T lymphocytes play a central role in vertebrate adaptive immune response. The Naïve T cells that are generated in the thymus mature into distinct subtype of T cells that differ greatly in their phenotypical and functional properties. Naive T cells when challenged with antigens undergo epigenetic alterations that affect expression of many genes involved in T cell-mediated immune responses. These changes ultimately lead to expression of cytokines that marks the functionality of T cells (1–4). Currently, the role of master regulators in the chromatin changes for lineage-specific differentiation of T cells is not well understood.

At the chromatin level, naïve T cell differentiation is associated with various changes in the nuclear matrix (4–6). A number of studies have suggested that the scaffold matrix attachment regions (SMARs) and groups of SMAR-associated proteins are required for transcription regulation at chromatin level during naïve T cell differentiation (7–9). For example, RUNX family of scaffolding proteins such as SATB1, CTCF, ID2, and BCL11b are known to associate with nuclear matrix and regulate gene transcription (10–17). However, it has been difficult to explain the defect observed in CD4^+^ T cell polarization after the loss of SMAR proteins. Although the significance of various signaling pathways toward CD4^+^ T cell differentiation have been studied extensively, reports suggesting the role of SMAR regions and SMAR proteins have been lacking.

Recent findings have suggested a role of transcription factors and nuclear matrix proteins in the development of auto-inflammatory disease including rheumatoid arthritis, experimental autoimmune encephalomyelitis (EAE), inflammatory bowel disease (IBD), and asthma (18–22). The manifestations of these diseases are correlated mainly by the disturbances in the conformation of chromatin that is facilitated by the nuclear matrix proteins (20, 23, 24). Perturbation in the chromatin conformation causes disturbances in the specificity of gene expressions. These abnormal gene expressions are the major cause of imbalance of CD4^+^ T cell response (25, 26). The exact mechanism by which the nuclear matrix proteins contribute to this lineage-specific gene expression in CD4^+^ T cells is not widely acknowledged. Thus, unraveling the nature and functions of these proteins assumes great importance in the current scenario of understanding the T cell biology and disease manifestations.

Here, we present a comprehensive study of nuclear matrix-binding protein SMAR1. SMAR1 through its DNA-binding ability acts as transcription regulator and chromatin modifier. It interacts with several key transcription factors like p53, NF-κB, and other chromatin regulatory factors that are involved in the regulation of many genes. Our recent findings suggest that SMAR1 is critical in regulating the fate of CD4^+^ T cell. It plays an important role in T cell development, differentiation, and proliferation by regulating plethora of genes. The essential role of SMAR1 in thymocyte development was established by studies using SMAR1 transgenic mice (27). SMAR1 transgenic mice display splenomegaly and enlarged lymph nodes with altered proportion of double negative (DN) thymocytes (27). Recently, our lab has suggested an essential role of SMAR1 in maintaining specific CD4^+^ T cell lineage fate during allergic and auto-inflammatory disorders. SMAR1 is essential for maintaining the lineage commitment between regulatory T (T_reg_) cells and other effector Th cells (Th1, Th2, and Th17 cells). T cell specific deletion of SMAR1 leads to altered immune response in allergic and auto-inflammatory diseases like asthma and colitis. Loss of SMAR1 in T_reg_ cells promotes re-differentiation of T_reg_ to other inflammatory Th cell lineage, which strongly suggests SMAR1 is involved in maintaining plasticity of T_reg_ cells. In this review, we focused SMAR1-mediated epigenetic regulation of T_reg_ and other effector T cell differentiation and their implications in modulating adaptive immune response during allergic and auto-inflammatory diseases.

## Nuclear Matrix-Binding Protein SMAR1: Essential Regulator of T Cell Development and Differentiation

**Figure 1:**
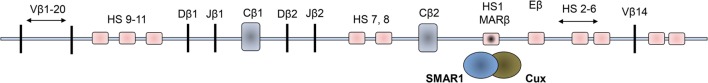
**Schematic representations of organization of the Vβ loci of the mouse double positive thymocytes**. The 11 hypersensitive sites (HS) are shown in light pink squared regions, and the dark squared region is the enhancer region where SMAR1 was found to bind to the *E*β.

Nuclear matrix proteins are integral part of the nucleus, which have a crucial role in the maintenance and stability of chromatin conformation that is necessary for the functionality of a particular cell (3). All the cellular processes in a cell are highly coordinated, which demand a correct orientation of chromatin domains (4, 28). Such an orderly arrangement is facilitated by the anchorage of specific sequences of the DNA to the nuclear matrix. This signature sequences known as SMARs serve as boundary elements that restrict the topology of chromatin to specific functional domains. Hence, proteins that have the ability to bind to these regions become important as they can govern the accessibility of activation/repression factors to the chromatin (29, 30). Abnormal levels of these proteins are observed in many disease conditions where extensive deregulation of gene expression occurs that signifies the role of these proteins in the regulation of genes. During the T cell development and differentiation, dramatic changes are happening at the chromatin, which involve major participation by the nuclear matrix proteins (31, 32). They are the major candidates for the chromosomal looping and interactions, which causes both intra- and interchromosomal interactions. The gene encoding for SMAR1 was identified from mouse T cell library and was initially considered to be thymus specific (Figure 1) (33). Further work into the functionality of SMAR1 highlighted considerable relevance in specific gene regulation (34, 35). Apart from its ability to anchor the chromatin to the nuclear matrix, SMAR1 can recruit chromatin modifying complexes such as HDAC1/SIN3, SMRT, and HDAC6 and regulate target gene expressions (34–37).

Scaffold matrix attachment region-binding protein 1 was identified in double positive thymocytes and described to have occupied in a MAR site within the *TCR*β locus. Binding of this protein regulates the V(D)J recombination and hence was assumed to be general regulator of gene transcription (38) (Figure 1). In the *TCR*β gene, Dnase hypersensitive sites (HS) were observed to be open in the DP stage of thymocyte development where SMAR1 was initially shown to attach with the DNA through the MAR regions (27). The binding of SMAR1 to the HS1 site near the *E*β enhancer was observed to reduce the *TCR*β rearrangement significantly. Overexpression of SMAR1 in the thymocytes exhibited reduced rearrangement of *TCR*β gene with elevated number of early DN thymocytes (27). Mice with overexpressed SMAR1 have perturbed immune responses, which confirm the immunomodulatory function of the protein. The T cells from SMAR1 transgenic mice exhibited a mild perturbation in the early DN stage. These mice also expressed altered frequency of T cells expressing commonly used *V*β*s* (27). These findings indicate the importance of SMAR1 in T cell development. T cell development in the thymus and its differentiation to various subsets coincide with chromatin changes. Studies on any cell intrinsic factors that regulate the fate of T cells thus have tremendous value in the medical research on different diseases. Thus, factors modulating the chromatin changes like nuclear matrix proteins assume to be of a significant importance in the development and differentiation of T cells.

### SMAR1 Is Critical for the Establishment of Th2 Phenotype

CD4^+^ T cell differentiation is a tightly controlled process requiring cytokine signaling pathways, which activates distinct transcription factors. During the course of this differentiation, several coordinated changes happen at the chromatin level leading to differential expression of genes specific to the functional aspects of the effector cells (39). Lineage-specific transcriptional factors and other chromatin proximal proteins interplay and mediate the activation of cytokine subsets marking a particular lineage commitment while repressing others (1, 40). Our lab provided the evidence that the expression of Th1-specific lineage commitment transcriptional factor T-bet could be regulated by SMAR1 and enhanced expression of SMAR1 caused defective Th1 response with a reciprocal increase in Th2 cell commitment (41). This inverse correlation of Th1/Th2 axis has been substantiated by many previous reports describing the differential function of proteins involved in the lineage specifications of T cell development (42, 43).

A large group of evidence has provided a clear insight into the involvement of chromatin changes associated with the naïve T cell differentiation into effector cells (44). IFN-γ and Th2 cytokine locus (IL-4, IL-5, and IL-13) undergo substantial changes in the chromatin conformation during Th1 and Th2 differentiation, respectively, orchestrated by interchromosomal and intrachromosomal interactions (45–47). These long range interactions and chromatin loop formations are consequence of temporal binding between the *cis* elements and many associated nuclear proteins (48–50). Many MAR-binding proteins are well characterized and described including CDP/Cux, SATB1, PARP, SAFs, and ARBP (30). Recently, a thymus-enriched MARBP, SATB1, has been shown to play a crucial role in the lineage determination and maintenance of Th2 (51, 52) and T_reg_ cells (53), respectively.

High throughput technologies including full genomic microarray has assisted the investigation and identification of many novel factors that are crucial for the differentiation of T cells (54, 55). Lineage-specific transcriptional factor T-bet induces the expression of IFN-γ through the chromatin remodeling of its gene along with CTCF and establishes a Th1 phenotype (56). Similarly, GATA3 induces chromatin changes at the Th2 locus and repressive changes at the IFN-γ locus (57). Thus, the function of lineage-specific factors and master regulators is to establish a particular lineage by inducing specific genes and at the same time repressing others (44). Many nuclear proteins such as IRF4 (58, 59), Gfi-1 (60, 61), Ikaros (62), and Dec 2 (9) have been documented to be selectively expressed in Th2 differentiated cells, and these proteins function either by upregulating the genes involved in the Th2 lineage commitment or by repressing the genes involved in the establishment of other cell lineages. We observed the role of SMAR1 particularly in the Th2 cells when its expression is selectively induced. In this condition, the expression of GATA3 is induced that results in activation of Th2 cytokine genes along with suppression of gene subsets that are committed to other lineages (63). Previous reports also suggested a reciprocal regulation of genes involved in the effector T cells differentiation (40), and we observed T-bet as a target of SMAR1 in Th2 differentiated cells. Our lab demonstrated an inverse correlation of T-bet expression in T cells from SMAR1 transgenic and SMAR1^−/−^ mice, showing the regulation of SMAR1 at the T-bet axis (41).

**Figure 2:**
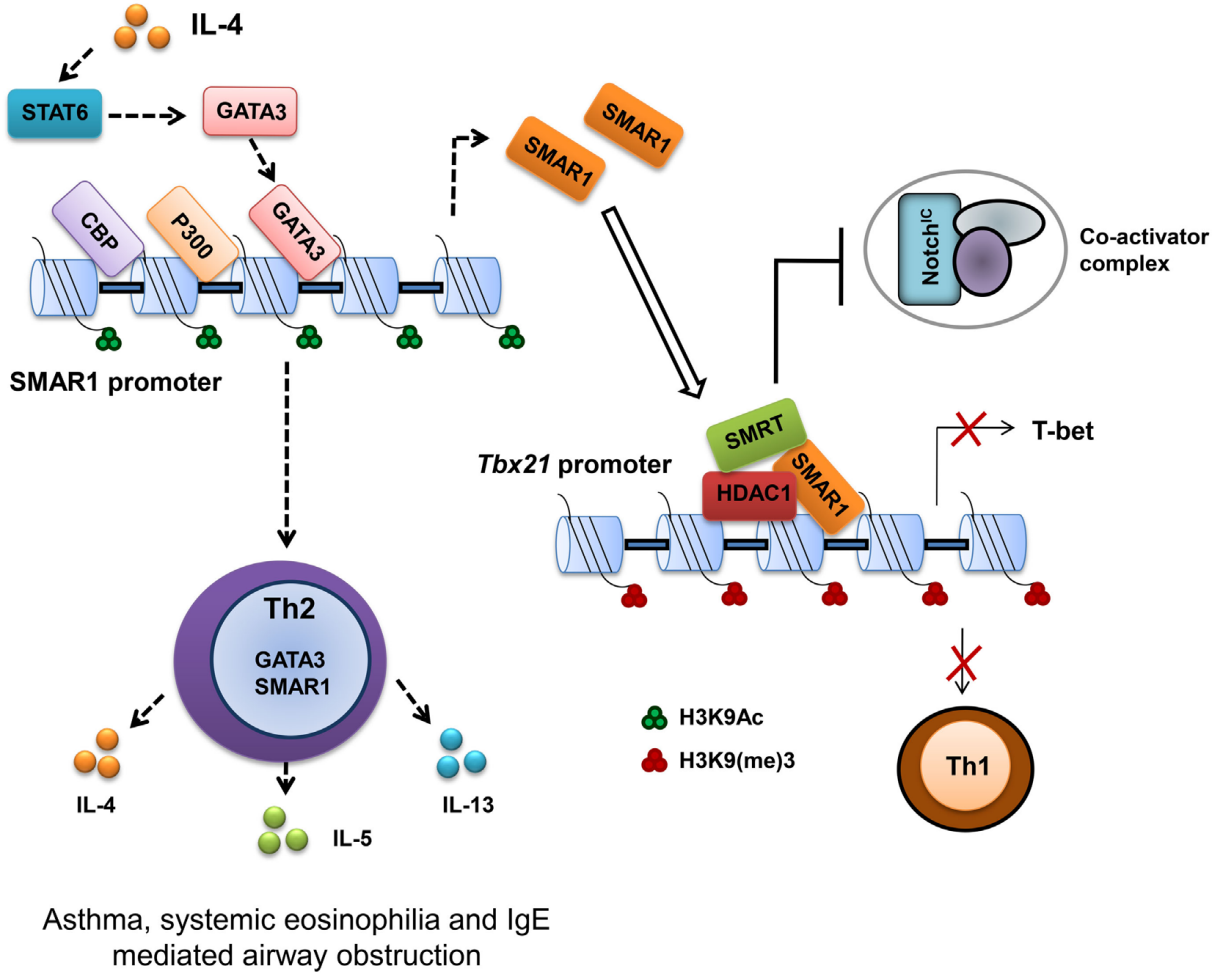
**Scaffold matrix attachment region-binding protein 1 (SMAR1) deficiency causes defective Th2 response both *in vitro* and *in vivo***. SMAR1 directly binds and forms a corepressor complex along with HDAC1/SMRT/RBP-Jκ and occupies CSL-binding consensus site on *Tbx21* promoter in turn preventing Notch1 (NICD) recruitment followed by T-bet transactivation. Enrichment of SMAR1 on *Tbx21* promoter changes the epigenetic signature of the target gene into a repressive mark with reduced acetylation and increased methylation of histones. This regulation of T-bet is crucial for the differential commitment of T cells to various lineages. Binding of SMAR1 on the *Tbx21* promoter decreases the transactivation of T-bet downstream IFN-γ. SMAR1 induction in Th2 cells is GATA3 dependent, and GATA3 directly binds and activates SMAR1 transcription. Here, we show that SMAR1 is a critical molecule in establishing the Th2 differentiation by controlling the transcription of Th1-specific factors like T-bet. Furthermore, we noticed in SMAR1^−/−^ mice under experimental induction of airway allergy, a defective Th2 response and reduced hyperresponsiveness.

T-bet is important for the differentiation of Th1 cells (64). Therefore, regulation of T-bet gene expression is important to establish Th1 and maintain Th1/Th2 axis as evidenced by the abnormal disease conditions correlated with the deregulation of T-bet (65). Previous studies on the regulation of T-bet promoter revealed an indispensable function of Notch in the transactivation of T-bet (66). Many putative cleaved activated Notch (CSL)-binding sites were characterized on the T-bet promoter crucial for the activation of T-bet in a Th1 specific condition. These binding sites function not only as enhancer elements but also as a regulatory region by an interplay of differential protein binding (67, 68). Notch1 activation is required for both Th1 (66) and Th2 cell lineage differentiation (68, 69), but SMAR1 is induced in Th2 differentiated cells. We noticed that GATA box-binding elements on SMAR1 promoter bind GATA3 and positively activate SMAR1 in Th2 differentiated condition. Furthermore, SMRT/HDAC complex has been demonstrated to mediate effective regulation at Notch target sites by functioning as corepressor (70). In agreement with these reports, we observed SMAR1-mediated downregulation of T-bet expression by directly interacting to the distal CSL-binding site on the T-bet promoter (41). In addition, the binding of SMAR1 to this region recruits the SMRT/HDAC corepressor complex on its promoter, and this corepressor complex competes with the Notch-mediated transactivation of T-bet in Th2 cells even at induced levels of Notch signaling (Figure 2). Moreover, the recruitment of corepressor complex modifies the chromatin at this region into a silencer mark by reduced histone acetylation and increased methylation. Thus, SMAR1 functions as an “adaptor molecule” crucial for the regulation of T-bet in Th2 cells at the chromatin level through differential binding to MAR sequences that mediates chromatin looping associated with necessary repressive modifications (41) (Figure 2).

T-bet expression drives aggressive and inflammatory processes by regulating such responses, which are essential for the prevention of organ specific autoimmunity (65). T-bet deficiency is correlated with increased hypersensitivity to allergen in airway (71). *Tbx21*^−/−^ CD4^+^ T cells showed Th2 biasness with signature hyper-acetylation of IL-4 promoter reflecting the suppressive effect of T-bet on the IL-4 locus (57). Moreover, T-bet over expression attenuates airway hypersensitivity by shifting the cytokine balance to Th1 response (65, 72). SMAR1^−/−^ mice showed a significantly reduced hypersensitivity response with lower frequency of IL-4-producing Th2 cells and eosinophilia in the BAL fluid. SMAR1^−/−^ mice were resistant to ovalbumin induced allergic airway inflammation. Since naïve CD4^+^ T cells from SMAR1^−/−^ mice have impaired Th2 differentiation in the lung, allergic inflammation leads to aberrant expression of genes that are responsible for Th1 and Th17 commitment, which in turn suppresses Th2 response *in vivo*. This observation is in line with the previous reports suggesting the elevated T-bet expression in SMAR1^−/−^ mice after chronic allergic antigen exposure (41, 71, 73). It shows SMAR1 is a novel and essential factor for the establishment of Th2 cells by functioning as a Th1-specific transcriptional gene repressor.

### SMAR1 Maintains of T_reg_ Phenotype and Controls of Inflammation

Regulatory T cells are central to controlling immune tolerance and maintaining immune homeostasis. Foxp3 is recognized as a single gene determinant essential for T_reg_ cell function (74). Alteration in Foxp3 expression, even the slightest, often leads to impaired T_reg_ cell function and is associated with various autoimmune and inflammatory disorders (75, 76). Many factors including Runx–CBFβ complexes, NF-κB, FOXO1, and FOXO3 are known to be important for Foxp3 expression (77). Deletion of either of these genes causes abrogation of Foxp3 expression. Other transcription factors have recently been shown to regulate Foxp3 expression including Bcl11b and TCF3 that bind to the Foxp3 promoter and induce its expression in response to TGF-β. TCF3 requires ID3 for GATA3-mediated repressive activity upon Foxp3 expression (78). Recent studies have revealed involvement of mTOR signaling pathways in the process of T cell fate determination, including the differentiation of naïve T cells into effector or T_reg_ cells. When activated, an mTOR-deficient T cell becomes Foxp3^+^ T_reg_ cells (79, 80). The main role of mTOR in regulating Foxp3^+^ T_reg_ cell responsiveness or stability has been implicated by studies using mTOR inhibitor, rapamycin (79). More recent studies of Rheb- or Rictor-deficient mice suggest that distinct mTORC1 or mTORC2 activities selectively regulate each subset of effector T cells, but inhibition of both is required for the spontaneous generation on Foxp3^+^ T_reg_ cells (81). Regarding the mechanism underlying the repression of Foxp3 in developing T_reg_ cells, IL-6- or IL-23-mediated activation of STAT3 has been shown to play a central role. STAT3 binds to CNS2 region of Foxp3 promoter and represses its expression (82). With regards to downstream pathways of STAT3, several genes including *Rora, Rorc, Batf, Irf4*, and *HIF-1*α have been demonstrated to be activated by STAT3 and are implicated in the Th17 cells differentiation (83, 84). Recent reports show a degree of plasticity through the acquisition of specific transcription factors in T_reg_ cell, which is required for controlling a defined polarized condition. In extreme inflammatory condition or in defined compartments, T_reg_ cells can also express effector cytokines (85, 86). For instance addition of RORγt in T_reg_ cell can produce IL-17A (85–87), indicating sustained expression of Foxp3 in T_reg_ cell is essential for maintaining its regulatory function.

Effector CD4^+^ T cells are responsible for the production of the pro-inflammatory cytokines that cause tissue damage. Conversely, T_reg_ cells are responsible for maintaining peripheral tolerance of effector T cells and keeping these cells in check (88). Our lab for the first has shown that the role of a MAR-binding protein, SMAR1 in maintaining the balance between Th17 and T_reg_ cells and its role in inflammatory diseases. In absence of SMAR1, T_reg_ cells lose their suppressive activity that leads to increased production of pro-inflammatory cytokine through T cells in the colon. These T cells showed upregulation of gut homing markers, integrin α4β7, and CCR9 that help them to accumulate in the gut during colonic inflammation. This observation revealed an indispensable role of SMAR1 in regulating T_reg_ cell function and immune tolerance that maintain the balance between T_reg_ and Th17 cells. Deletion of SMAR1 in T cells enhances Th17 cells activity in experimental colitis, and the increased number of Th17 cells is thought to be the reason for the progression of the disease (89, 90). SMAR1-deficient T_reg_ cells are not able to prevent IBD in *Rag*^−/−^ mice, indicating that the suppressive function of T_reg_ cell is severely compromised. However, SMAR1-deficient T_reg_ cells showed reduced levels of IL-10 and upregulation of pro-inflammatory cytokines, including TNF-α, IL-17, and IFN-γ (89). Studies have shown that IL-10-deficient mice lack T_reg_ cells and are not capable of controlling inflammatory responses in the intestine (91, 92). In the absence of SMAR1, T_reg_ cells fail to suppress the reactive CD4^+^ T cells, as a result the whole balance among CD4^+^ T cells is severely damaged leading to the progression of the disease (89, 90, 93).

**Figure 3:**
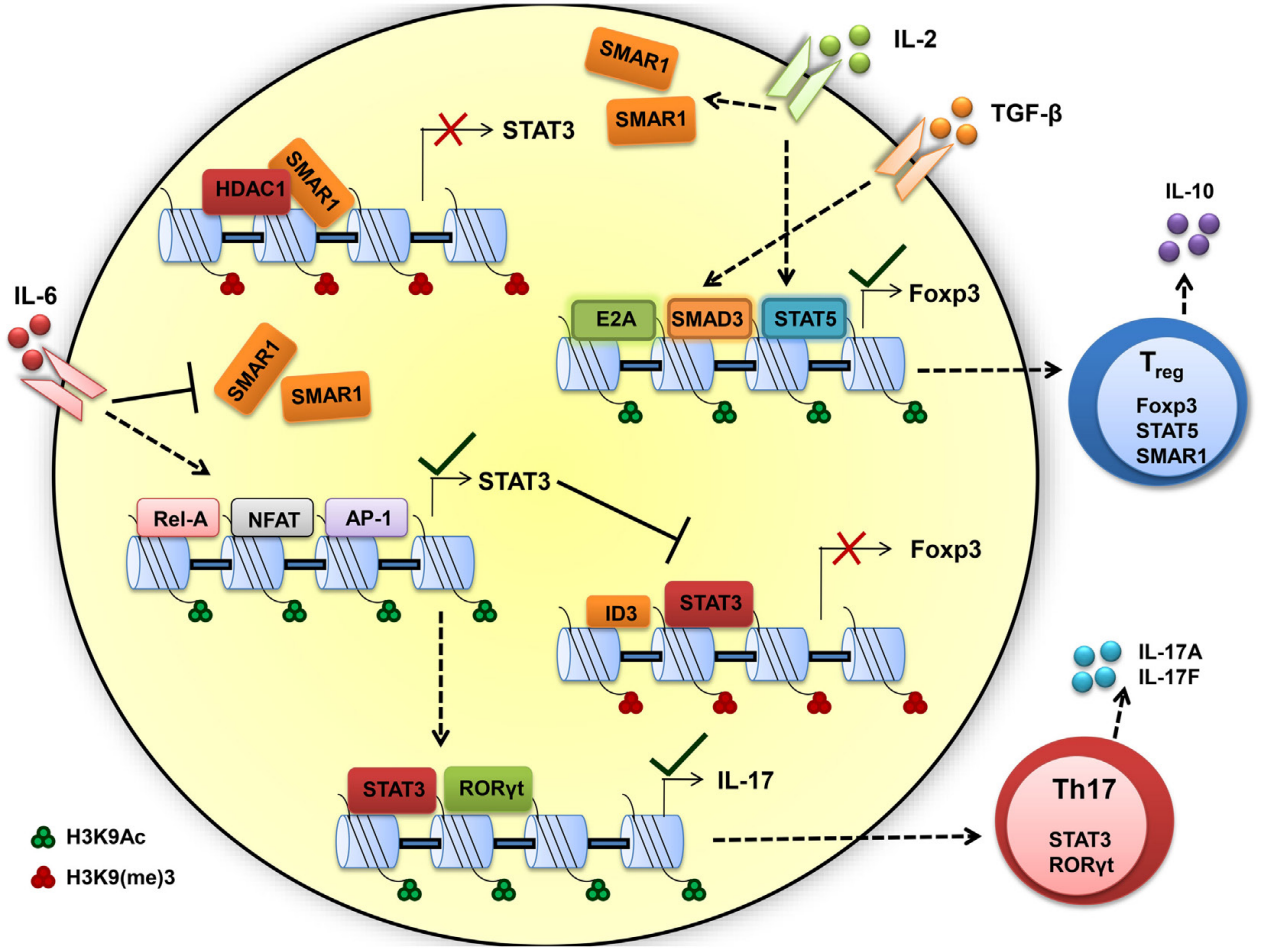
**Scaffold matrix attachment region-binding protein 1 (SMAR1) is required to maintain high Foxp3 expression and regulatory T (T_reg_) phenotype**. TGF-β and IL-2 signaling upregulate the SMAR1 expression that induces Foxp3 expression, by promoting SMAD3/STAT5 binding to the Foxp3 promoter to positively regulate transcription and by repressing the negative factor such as STAT3 and maintaining T_reg_ phenotype. In presence of inflammatory cytokine IL-6, SMAR1 expression is downregulated and STAT3 expression is upregulated, consequently STAT3 bind to CNS2 region of Foxp3 promoter in CD4^+^ T cells and represses Foxp3 expression. This fate attributes to more IL-17 production by positive regulation of IL-17 *via* STAT3 and RORγt, a Th17 lineage-specific transcription factors. Thus, SMAR1 is required for the control of STAT3 production and consequent IL-17 expression in Foxp3^+^ T_reg_ cells. The important activity that define the Th17 and T_reg_ function are indicated by arrow, dotted arrow indicate upregulation whereas plain arrow indicate downregulation.

We found constitutive expression of SMAR1 in natural T_reg_ cells as well as induced T_reg_ (iT_reg_) cells. Data from our lab support the idea that IL-2 contributes to the expression of SMAR1 through T_reg_. Recent reports suggest that the T_reg_ cell require acquisition of specific transcription factors to exhibit control in defined polarized situation (94, 95). Previous reports demonstrated that increased expression of RORγt in T_reg_ can produce IL-17A (85) that leads to compromised T_reg_ functions. We have shown that in the colon, expression of RORγt was influenced by SMAR1 in T_reg_ and increased expression of IL-17A compared to WT (89, 93). The expression of Foxp3 is reduced by genetic alteration causing upregulation of RORγt, followed by increased levels of IL-17A production and generation of effector Th17 cells (86, 87, 96). Therefore, the loss of SMAR1 has severe loss on Foxp3 expression, leading to the induction of IL-17 conferring a T_reg_ phenotype to Th17 phenotype (Figure 3).

Understanding of how SMAR1 regulates immune function is still unclear. Indeed, our work opens a new role of SMAR1 in controlling T_reg_ cell function. The predominant cell type that expresses Foxp3 is CD4^+^CD25^+^ T cell; the same population that has been reported to suppress proliferation and cytokine production in conventional CD4^+^ T cells (97). Foxp3 appears to function through the transcriptional repression of many genes including the effector cytokines (98, 99). The factors that mediates the trans-activation or trans-repression are critical to delineate the molecular mechanisms involved in controlling regulation of Foxp3. Previous reports suggest that TGF-β mediates enrichment of SMAD2/3 at the Foxp3 promoter and the activation of Foxp3 transcription (100, 101). On the other hand, STAT3 is reported to bind to silencer regions of Foxp3 promoter (100, 102) and suppresses its expression. Deficiency of SMAR1 in T_reg_ cells leads to uncontrolled STAT3 production and results in the production of IL-17. Additionally, IL-6-mediated suppression of SMAR1 has a direct effect on the enrichment of STAT3 at Foxp3 promoter. Inhibition of SMAR1 restores STAT3 enrichment in Foxp3 promoter in response to TGF-β1 in SMAR1^−/−^ CD4^+^ T cells (89). Finally, IL-6 influences Foxp3 epigenetically by loosening the chromatin allowing the access of STAT3 to the Foxp3 promoter. These observations support the idea that over expression of STAT3 is a key factor in defective Foxp3 induction in SMAR1^−/−^ CD4^+^ T cells. It is known from earlier studies that SMAR1 affect transcriptional activity mainly through DNA binding (34, 35). Presence of several MAR-binding regions at the promoter of STAT3 suggests that SMAR1 can potentially bind to these sites and influence the STAT3 expression. SMAR1 bound to regulatory regions of STAT3 locus could inhibit the activity of STAT3, a negative regulator for Foxp3 (103, 104) (Figure 3). In support of this idea, we observed lower expression of SMAR1 in Th17 cells (41) and was unable to bind to STAT3 locus. However, in T_reg_ cells, SMAR1 binds at a position -660 to -840 associated with strong MAR and -229 to -478 associated with IL-6 response elements from the transcription start site of STAT3 locus (89). Our lab showed that in the WT cells treated with TGF-β1, SMAD2/3 bind to the Foxp3 promoter. At the same time, SMAR1 was found to bind STAT3 promoter suggesting a positive role for SMAR1 in the transcriptional regulation of STAT3. This activity was regulated through TGF-β signaling. This observation also suggests a role of SMAR1 in regulating Foxp3 expression in TGF-β1 iT_reg_ cells and thus SMAR1 ultimately decides the plasticity of T_reg_ cells (Figure 3).

Reports on plasticity of T_reg_ cells in inflammatory response showed the control of T_reg_ cells by specific transcription factors in polarized condition, and loss of this polarity leads to expression of effector cytokines (42, 87). We found that expression of SMAR1 in T_reg_ cells is downregulated during colonic inflammation and SMAR1-deficient T_reg_ cells produced large amount of pro-inflammatory cytokine IL-17 compared to WT. This clearly shows effector cytokine production by T_reg_ cells occurred in a condition in which SMAR1 was either reduced or absent (93).

We also assessed the relative contribution of IL-10 in mediating T_reg_ cell immunosuppressive function. Neutralization of IL-10 in SMAR1^−/−^ mice led to an interesting finding that compensation for T_reg_ cell defects also depend on IL-10 signaling. Foxp3 amount in SMAR1^−/−^ mice is greatly diminished upon anti-IL-10 treatment following DSS administration; we demonstrated that T_reg_ cells are a major source of IL-10 in SMAR1^−/−^ mice. It is proposed that IL-10-secreting T_reg_ cells are a critical component of immune-mediated protection during increased intestinal inflammation in SMAR1^−/−^ mice (93). In the context of T_reg_ cell biology, the current study reveals a novel role of SMAR1 in controlling T_reg_ physiology during inflammation. Therefore, to study the factors that are modulating the regulation and function of T_reg_ is an interesting target for immunotherapy in inflammatory disorders.

## Therapeutic Applications of SMAR1

### The Implications of SMAR1 Nanotherapy for the Treatment of Auto-Inflammatory Diseases

**Figure 4:**
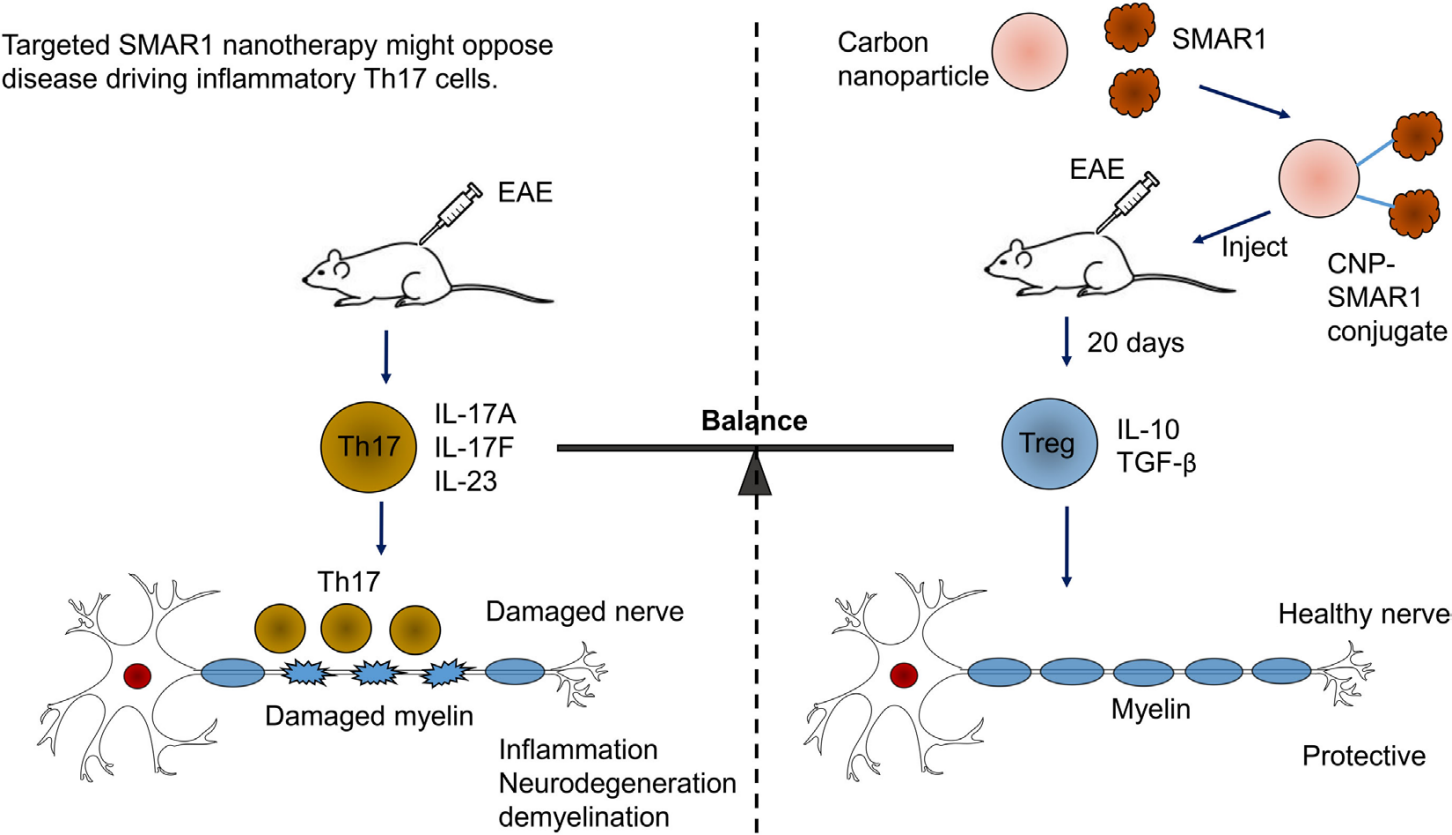
**Targeted scaffold matrix attachment region-binding protein 1 (SMAR1) nanotherapy might oppose disease driving inflammatory Th17 cells**. Conjugation of carbon nanospheres (CNPs) with SMAR1 provides a sustained delivery of SMAR1 over several days, allowing time for epigenetic stabilization of anti-inflammatory regulatory T (T_reg_) cells. Since T_reg_ cells releases anti-inflammatory cytokines, the effect of CNP-SMAR1 will become sustained by endogenous as well as induced T_reg_ cells that restrain myelin affecting Th17 cells. Thus, CNP–SMAR1 provides a distinct way to treat experimental autoimmune encephalomyelitis (EAE) by regulating two endogenous pathways, one suppressing pathogenic Th17 cells, the second allowing T_reg_ cells generation those are protective and control the inflammation.

Scaffold matrix attachment region-binding protein 1 has emerged as important factor for gene expression by regulating epigenetic modifications. SMAR1 seems to coordinate cytokine-dependent gene expression in CD4^+^ T cells. We have shown SMAR1 is downregulated under Th1 and Th17 differentiation, and we have observed IL-6, a major cytokine involved in generation of Th17 cells, downregulates SMAR1 expression (89). Our lab has also reported that SMAR1 regulates STAT3 gene expression by directly binding to STAT3 promoter and recruiting the repressor complex (89). In agreement with this, a deficiency of SMAR1 in T cells renders the mice susceptible to myelin oligodendrocyte glycoprotein peptide-driven EAE disease with higher pro-inflammatory IL-17-producing T cells (105). EAE is an animal model of human CNS demyelinating disease, including multiple sclerosis (MS). Researches have shown that IL-17-secreting Th17 cells are the causative mediator of the disease. Thus, EAE is considered to be Th17-driven auto-inflammatory disease (106). EAE disease progression can be controlled by signaling molecules or transcription factor that prevents Th17 generation (106, 107). We have shown using a nanoparticle-mediated delivery that SMAR1 controls the Th17 generation and EAE disease progression (105). The small size and high surface volumes of nanoparticles makes them a convenient route of drugs/proteins delivery inside the cell (108). Currently, diverse types of nanoparticle including carbon-based nanoparticles, metal-based nanoparticles, and dendrimers are used for protein delivery (109, 110). We used carbon nanospheres (CNPs) as a carrier for delivery of SMAR1 at the site of inflammation. We observed CNP-mediated delivery of SMAR1 represses the EAE disease progression by inhibiting the IL-17 expression from T cells (Figure 4). Recent study from our lab has shown that nanoparticle-mediated SMAR1 delivery could potentially be used to suppress auto-inflammatory diseases (105). As a treatment for MS, CNP-SMAR1 has three therapeutic values (i) opposition of Th17 differentiation, (ii) increment in the anti-inflammatory IL-10 production by favoring T_reg_ differentiation, and (iii) promotion of the self-tolerance to myelin. Controlling inflammation by treating inflammatory Th17 cells during EAE by CNP-SMAR1 provides a virus and drug free option to current strategies of MS treatments (Figure 4).

### SMAR1 in Diagnosis and Treatment of Inflammatory Diseases like IBD

Abnormal inflammatory responses cause many adverse effects to the body as observed in many disease conditions. Pro-inflammatory Th1 and Th17 cells are attributed to many of these disease conditions and targeting the pro-inflammatory cells is now assumed to be a pivotal option. Most therapies in autoimmune and inflammatory disorders are aimed at general supersession of the inflammatory responses (111, 112). Since autoimmune and inflammatory disorders are the result of an imbalance in immune regulation, a different approach that modulates T_reg_ population could potentially be a target (113, 114). The main strategy in treating IBD is to halt the ongoing inflammation and prevent permanent tissue damage. T_reg_ cells play a key role in regulation of IBD, and recently, the number of studies has described the presence and function of T_reg_ cells in patients with IBD (115, 116). In the colon, a target organ of IBD, T_reg_ cells, was shown to be decreased by 40–50% compared to peripheral blood from IBD patients (117, 118). Therefore, regulating the functions of T_reg_ is an interesting target for immunotherapy in IBD. We elaborate the role of MARs and SMAR1 in CD4^+^ T cell gene regulation by altering the local chromatin structure that governs the Foxp3^+^ T_reg_-mediated immune response. Therefore, not only T cell-modulating cytokine but also MARs and MAR-binding proteins such as SMAR1 could be an interesting target to reduce pro-inflammatory IFN-γ- and IL-17-producing Th1 and Th17 cells. We have shown that SMAR1 regulates some essential genes that dictate the CD4^+^ T cell phenotype and that the SMAR1 aberrant expression leads to dysregulated T cell polarization. SMAR1 level gradually decreases during the development of auto-inflammatory disorders (90, 93), it can be therefore used as a marker for diagnosis of T cell-mediated auto-inflammatory disorders. Identifying the epigenetic modifications of SMAR1-targeting pro-inflammatory cytokine genes in T_reg_ cells leads to its role as a potential candidate for the use as anti-inflammatory drugs. Though the studies so far have elucidated the role of SMAR1 with respect to tumor suppressor, our recent studies initiated to establish the anti-inflammatory function of SMAR1 in autoimmune disorders like EAE and IBD.

## Conclusion

Various MAR-binding nuclear proteins are involved in crosstalk between genetic and epigenetic factors during differentiation of naïve T cells through chromatin changes. Studying “adaptor proteins” that bind to chromatin and define chromatin conformation provides us with cues to understand the mechanism of T cell differentiation. In this review, we described the indispensable role of one such MAR-binding protein, SMAR1, in regulating distinct subsets of gene during T cell differentiation and perturbed immune responses correlated with deregulation of SMAR1. We also addressed the possible molecular mechanism involved in the gene transcription in the context of chromatin changes during CD4^+^ T cell differentiation. Further investigation into the possibilities of identifying novel molecular targets will be beneficial in modulating therapeutic interventions and immune responses.

## Future Perspectives

The function of SMAR1 in T helper cell differentiation is crucial as described in this review. However, the role of SMAR1 in memory T cell differentiation and maturation are not studied in detail and require further investigation. Since SMAR1 regulates genes that are essential for specific T cell lineage commitment, it is also important to examine whether SMAR1 plays a role in differentiation of Th9 or Th22 cells, a novel CD4^+^ T cells subsets. Findings from recent studies have emphasized the requirement of SMAR1 in controlling the expression of STAT3 during T_reg_ differentiation. It would be interesting to study the regulation of SMAR1 in T_reg_ cells that could be regulated by an IL-6:STAT3 or IL-2:STAT5 dependent mechanism as STAT3 and STAT5 are essential transcription factors required for Th17 and T_reg_ differentiation, respectively. It would be also exciting to investigate whether SMAR1 play a role in the T follicular helper cell differentiation. Studies illuminating the role of lincRNAs in the regulation of SMAR1 in CD4^+^ T cell subtypes could also elucidate the signaling pathways and molecular mechanisms that regulate the lineage commitment of various subtypes of CD4^+^ T cells.

## Author Contributions

BM, DG, and SC contributed to the manuscript text; BM prepared the figures; and BM, DG and SC conceived the structure of the review.

## Conflict of Interest Statement

The authors declare that the research was conducted in the absence of any commercial or financial relationships that could be construed as a potential conflict of interest.
